# Local Population Characteristics and Hemoglobin A1c Testing Rates among Diabetic Medicare Beneficiaries

**DOI:** 10.1371/journal.pone.0111119

**Published:** 2014-10-31

**Authors:** Laura C. Yasaitis, Thomas Bubolz, Jonathan S. Skinner, Amitabh Chandra

**Affiliations:** 1 Harvard Center for Population and Development Studies, Harvard University, Cambridge, Massachusetts, United States of America; 2 The Dartmouth Institute for Health Policy and Clinical Practice, Geisel School of Medicine at Dartmouth, Hanover, New Hampshire, United States of America; 3 Department of Economics, Dartmouth College, Hanover, New Hampshire, United States of America; 4 The John F. Kennedy School of Government, Harvard University, Cambridge, Massachusetts, United States of America; CUNY, United States of America

## Abstract

**Background:**

Proposed payment reforms in the US healthcare system would hold providers accountable for the care delivered to an assigned patient population. Annual hemoglobin A1c (HbA1c) tests are recommended for all diabetics, but some patient populations may face barriers to high quality healthcare that are beyond providers' control. The magnitude of fine-grained variations in care for diabetic Medicare beneficiaries, and their associations with local population characteristics, are unknown.

**Methods:**

HbA1c tests were recorded for 480,745 diabetic Medicare beneficiaries. Spatial analysis was used to create ZIP code-level estimated testing rates. Associations of testing rates with local population characteristics that are outside the control of providers – population density, the percent African American, with less than a high school education, or living in poverty – were assessed.

**Results:**

In 2009, 83.3% of diabetic Medicare beneficiaries received HbA1c tests. Estimated ZIP code-level rates ranged from 71.0% in the lowest decile to 93.1% in the highest. With each 10% increase in the percent of the population that was African American, associated HbA1c testing rates were 0.24% lower (95% CI −0.32–−0.17); for identical increases in the percent with less than a high school education or the percent living in poverty, testing rates were 0.70% lower (−0.95–−0.46) and 1.6% lower (−1.8–−1.4), respectively. Testing rates were lowest in the least and most densely populated ZIP codes. Population characteristics explained 5% of testing rate variations.

**Conclusions:**

HbA1c testing rates are associated with population characteristics, but these characteristics fail to explain the vast majority of variations. Consequently, even complete risk-adjustment may have little impact on some process of care quality measures; much of the ZIP code-related variations in testing rates likely result from provider-based differences and idiosyncratic local factors not related to poverty, education, or race.

## Introduction

New payment models, including Accountable Care Organizations (ACOs), create incentives for providers to deliver high quality care to all patients attributed to them. Yet, some patient populations may face barriers to high quality care beyond the control of healthcare providers. Transportation costs, and time or financial constraints, may differ across demographic subpopulations, perhaps explaining part of measured care disparities among vulnerable populations. Additionally, the local environment in which a person lives may have a strong effect on his or her ability to seek care [1], [2]. If such effects are present, payment mechanisms may need to take them into account to prevent ACOs from avoiding such patients, or from being penalized with lower reimbursements [3], [4], thus potentially worsening current disparities. In fact, recent draft recommendations from the National Quality Forum suggest adjusting for sociodemographic factors in some quality measures used in pay-for-performance contracts, due to these concerns [5].

Current studies of healthcare quality and disparities have focused mainly on providers or large geographical areas [6]–[11]. Traditional approaches of aggregating population data to large areas – whether states, counties, or Hospital Referral Regions (HRRs) – likely obscure any potential local effects on residents' care. Healthcare providers, especially emerging ACOs, and the payers working with them, are affected by granular variations in healthcare quality that may vary from one ZIP code to the adjacent one. If characteristics of the local area are strongly related to adherence and testing measures, payment mechanisms may need to be adjusted to appropriately reward care for identifiable vulnerable populations. Alternatively, if living in a specific region has an independent effect on residents' healthcare experiences, beyond that expected by the composition of the local population, providers should be aware of local barriers to care and seek ways to minimize them.

To date, there have been no fine-grained measures available to study the contribution of these sociodemographic factors to variations in healthcare quality in the US. In this paper, we develop ZIP code-level measures of HbA1c testing rates during 2008–2010 among diabetic Medicare beneficiaries. We then compare these rates to local population demographics from the US Census (2010) and American Community Survey (2006–2010), and assess the proportion of variation in testing rates that can be explained by local population characteristics.

## Methods

### Data Sources

We examined Medicare claims from a 20% national sample of the Denominator, Medpar, Carrier, and Outpatient files for the years 2008, 2009, and 2010. Claims were linked to the ZIP code of residence provided for each beneficiary. Population statistics at the level of the ZIP code tabulation area (ZCTA) came from 2010 Census data and from census tract-level American Community Survey data (pooled across 2006–2010) that had been aggregated to the ZIP code-level. ZIP code-level population measures included total population, overall population density, and the percent African American (all from Census 2010), as well as the percent living below 100% of the federal poverty level (FPL) and the percent with less than a high school education (all from the American Community Survey).

### HbA1c Testing Rate Data

Healthcare Effectiveness Data and Information Set (HEDIS) definitions were applied to Medicare claims to select diabetic beneficiaries for two separate cohorts; the first defined for the years 2008–2009, and the second for the years 2009–2010. Two separate cohorts were created so that we could estimate spatially smoothed rates using one year's data, and validate the estimates with data from a different sample. To be included, a beneficiary had at least one acute inpatient or emergency department encounter, or two ambulatory or non-acute inpatient encounters, accompanied by a diabetes diagnosis, over the two-year period [12]. The outcome, receipt of an HbA1c test, was determined by at least one valid claim indicating such a test (CPT codes 83036 or 83037) in the second year of the cohort period (2009 or 2010). Current diabetes care guidelines suggest that patients receive HbA1c tests at least annually to help guide treatment decisions [13].

Age at the beginning of the observation period, gender, and self-reported race were recorded from the Denominator files. Each cohort was limited to beneficiaries covered under fee-for-service Medicare and aged 65 to 75, as those under 65 are likely to be systematically different from older beneficiaries, and current HEDIS specifications recommend these measures only up to age 75 [12]. We also excluded beneficiaries with any visits to Federally Qualified Health Centers or Rural Health Centers during the two-year observation period, as such visits are reimbursed with a single flat fee; specific services such as HbA1c tests are less likely to be recorded.

### Longitudinal ZIP Code Files

To match Medicare beneficiaries to physical locations, an assignment file mapped every recorded ZIP code since 1990 to the physical location of a 2010 ZIP code. (Older ZIPs may be required if a Medicare enrollee had not updated his or her mailing address.) In some cases for the earlier years, there was no exact numerical match to a 2010 ZIP code, so the older ZIP was assigned to its nearest physical neighbor in 2010, resulting in a final dataset in which all available ZIP codes from 1990 to present were assigned a physical location.

### Spatial Analysis

Analysis of geographic healthcare data often relies on aggregation to areas such as counties, states, metropolitan statistical areas, Hospital Service Areas (HSAs) or Hospital Referral Regions (HRRs) [7], [14]–[17]. Aggregation implicitly assumes that all the area's residents can be represented by the same estimate. Additionally, changes in the shape and size of the regions can affect the estimates recorded for those regions [18]. These concerns led us to pursue a spatial smoothing technique to explore fine-grained variations in HbA1c testing rates. ZIP codes were used because they were the finest grained geographical area available in Medicare claims.

Spatial smoothing approaches have been used previously to develop regional estimates of infrequent disease or health events [15],[19]–[21]. These methods take advantage of spatial autocorrelation – closer regions are more alike than those further away [22] – by borrowing information from a point's neighbors to adjust the estimate of a value for which there may be uncertainty. In this study, we used a disc smoother with an adaptive radius; the radius expands and contracts as necessary to ensure each estimate is based on a population above a minimum threshold [20]. We selected a population threshold of 50 beneficiaries for each rate estimate.

This spatial smoothing approach was applied to the 2008–2009 cohort of diabetic Medicare beneficiaries. The smoothing process was carried out iteratively. If a ZIP had greater than 50 diabetic beneficiaries, that population's unadjusted rate was assigned to the ZIP. If it had fewer, the next nearest ZIP was included, until the total population was at least 50; the population-weighted average across the entire set of ZIPs was then assigned to the central point.

### Testing Rate Estimate Validation

The estimates created using the 2008–2009 cohort were validated using the 2009–2010 cohort; a different cohort was used simply to allow for out-of-sample validation, rather than re-using the same data from which the estimates were created. All the ZIP codes with data from both periods were sorted into beneficiary-weighted deciles based on the HbA1c testing rate estimates from 2009. We then assessed the mean and interquartile range of the raw (unsmoothed) testing rates observed in 2010 for each decile of ZIP codes. As the raw rates from 2010 were based on as few as a single beneficiary, they were quite variable. Yet, this comparison allowed us to assess whether the spatial smoothing process retained much of the same overall information present in the raw rates.

### Statistical Analyses

HbA1c testing rate estimates were matched to population data from the US Census and ACS. ZIP codes that had both testing rate and population data were sorted into beneficiary-weighted deciles of the spatially smoothed estimated HbA1c testing rates. To compare the general population characteristics in high- and low-rate ZIP codes, we determined the average percent across deciles that was African American, had less than a high school education, or was living below 100% FPL. Deciles were used because they allow for comparison across several meaningful cut-points (e.g. lowest or highest 10%, median), yet maintain a manageable number of groupings for presentation and interpretation.

To assess the proportion of variance in ZIP code testing rates explained by demographic characteristics, we performed a ZIP code-level regression of raw testing rates on local population characteristics: population density, the percent African American, the percent with less than a high school education, and the percent living below 100% FPL. For the regression analyses only, we used raw testing rates due to concerns that our spatial smoothing process may have introduced excessive spatial autocorrelation into our dependent variable. If there were excessive spatial autocorrelation in the dependent variable, then it may be possible to detect spurious relationships between that variable and independent variables that also display spatial autocorrelation; standard errors would likely be artificially small as well. Population density was represented with decile indicators to capture potential non-linear effects (exploratory analyses using the other covariates did not suggest strong non-linear relationships). We weighted this regression by the number of diabetic Medicare beneficiaries in each ZIP in order to address concerns of statistical noise when the outcome was based on small populations.

We examined geographic variation in testing rate estimates by creating maps at the national and regional levels. Four sets of regional maps were created using geographic ZIP boundaries downloaded from ArcGIS. These maps depict ZIP code population characteristics and estimated HbA1c testing rates for Los Angeles, Houston, and Chicago.

### Sensitivity Analysis

We used raw testing rates in our regression analysis due to concern that excessive spatial autocorrelation introduced into our dependent variable in the smoothing process would result in spuriously small standard errors [23]. Yet, raw rates may be unstable; we therefore repeated this analysis among just those ZIP codes whose rates were derived from a minimum number of Medicare beneficiaries (5, 10, or 25), as well as with the smoothed estimates as the dependent variable.

ArcGIS 10 software was used to create geographic data files from latitude and longitude information for all ZIP codes, as well as representative maps of the final estimates. GeoDa software was used to define the nearest 100 neighbors for each ZIP. A custom program written in STATA 11 was used to complete the iterative smoothing process by combining the nearest neighbor files with initial ZIP code-level data derived from Medicare claims.

### Role of the Funding Source

This work was funded by the National Institute on Aging. The funding source had no role in the study design, conduct, and analysis or in the decision to submit the manuscript for publication.

### Human Subjects Protection

The work on this study was approved by the Dartmouth College Committee for the Protection of Human Subjects. Consent for the use of beneficiaries' claims in this study was waived, as the work consisted of secondary analysis of existing data, and all data were anonymized before any analysis was performed.

## Results

Table 1 displays general summary statistics from the Medicare beneficiary cohort and their distribution across ZIP codes of residence. Of 480,745 beneficiaries identified during the period of 2008–2009, 13.3% were African American, and about half (50.6%) were female. The beneficiaries resided across 29,438 different ZIP codes; of these, 4,818 (16.4%) had only a single beneficiary, while 6,729 (22.8%) had at least 25 beneficiaries, a common minimum population for public reporting [24]. The overall HbA1c testing rate among all beneficiaries was 83.3%. Of the 480,745 beneficiaries, 469,115 (97.6%) were successfully merged to ZIP-level population data from 25,190 residential US ZIP codes, in which resided about 98.7% of the general US population in 2010.

**Table 1:** Characteristics of diabetic Medicare beneficiary cohort and their ZIP codes of residence.

Beneficiary characteristics	
Total	480745 (100%)
African American	63986 (13.3%)
Non-African American	416759 (86.7%)
Mean Age	70.3
% Female	50.6%
% Received A1c	83.3%
Distribution of ZIP code-level A1c testing rate estimates	
Mean	83.3%
5^th^ percentile	71.7%
25^th^ percentile	79.6%
Median	84.1%
75^th^ percentile	88.1%
95^th^ percentile	92.9%
Distribution of number of diabetic Medicare beneficiaries per ZIP	
Total (N)	29438
1 beneficiary only	4818 (16.4%)
ZIPs with> = 5 beneficiaries	17417 (59.2%)
ZIPs with>25 beneficiaries	6729 (22.8%)

Source: Authors' calculations from Medicare claims data, 2008–2009.

**Figure 1:**
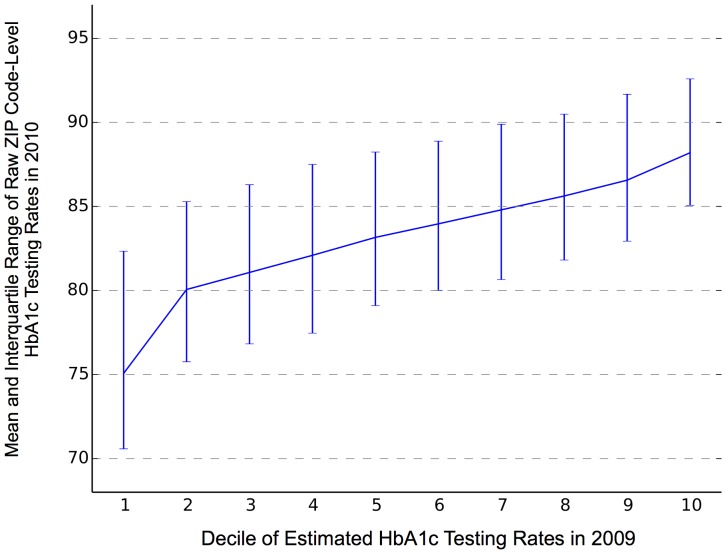
Mean and Interquartile Range of Raw HbA1c Testing Rates in 2010, by Decile of Estimated Rates in 2009.

We first validated the spatially smoothed ZIP-level estimates by comparing them to the raw rates for the following year. The ZIP codes with rates from both years were divided into deciles based on the 2009 smoothed rates. As the raw rates can be based on as few as 1 beneficiary, the minimum and maximum in each decile ranged from 0–100%. In Figure 1, we present the beneficiary-weighted mean and interquartile range of raw 2010 rates for each decile of 2009 rate estimates. The mean of the 2010 raw rates was 75.1% in the lowest decile, and rose steadily to 88.2% in the highest decile.

To compare local testing rates and associated population demographics, we divided the ZIP codes with both estimated rates and population data into deciles of 2009 estimated HbA1c testing rates. In Table 2, we present population-weighted summary statistics from the 2010 Census or 2006–2010 American Community Survey for each of these deciles. The mean estimated HbA1c testing rate was 71.0% in the lowest decile, and increased to 93.1% in the highest decile. The percent of the general population that was African American decreased from 19.9% in the lowest quality decile to 8.5% in the highest. The percent of the general population with less than a high school education, as well as the percent living below 100% FPL, also decreased as quality scores increased, from 11.7% to 5.1% and from 19.0% to 11.4%, respectively.

**Table 2:** Summary Statistics Across Deciles Of Estimated ZIP Code-Level HbA1c Testing Rates.

Decile of quality	1(low)	2	3	4	5	6	7	8	9	10(high)	Ratio Lowest to Highest decile
Average ZIP-level HbA1c testing rate	71.0	77.0	79.6	81.6	83.3	84.8	86.3	87.9	89.8	93.1	0.76
Average ZIP % African American 2	19.9	18.2	16.3	14.8	13.0	12.9	11.2	10.7	9.9	8.5	2.34
Average ZIP % with less than HS educ 2	11.7	9.1	8.3	7.1	7.1	6.5	6.2	5.9	5.4	5.1	2.29
Average ZIP % below 100% FPL 2	19.0	16.3	14.9	14.2	13.4	12.7	12.6	11.7	11.6	11.4	1.67
Number of ZIPs 1	2643	2438	2346	2464	2384	2380	2653	2483	2674	2725	

Quality deciles reflect beneficiary-weighted HbA1c testing rate deciles. All mean estimates are weighted by total US population in each ZIP code.

1 ZIP codes with estimates of HbA1c testing rates (from Medicare claims) and demographic data.

2 Data from US Census and American Community Survey.

**Figure 2:**
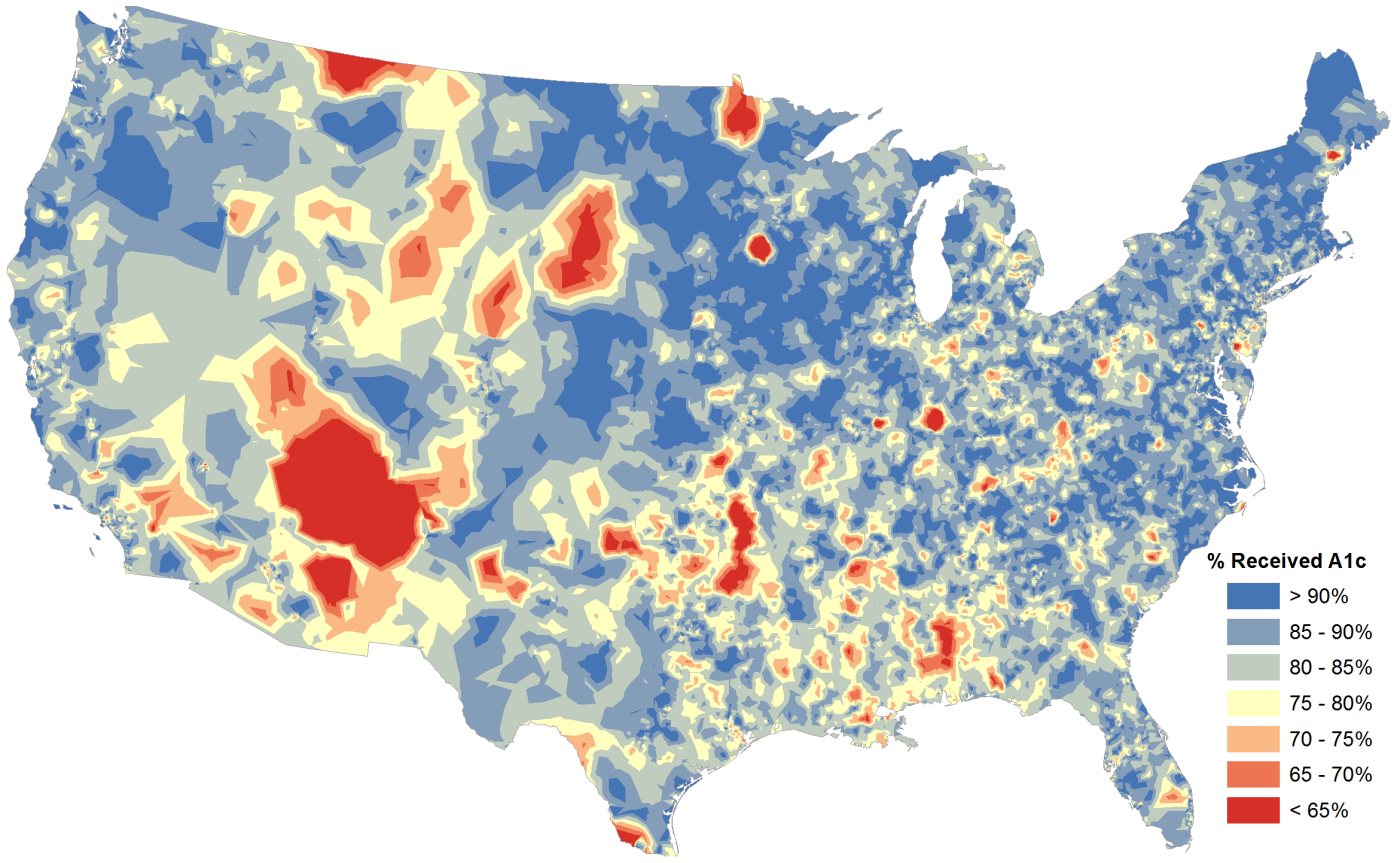
National Map of Estimated HbA1c Testing Rates Among Medicare Beneficiaries, 2009.

**Figure 3:**
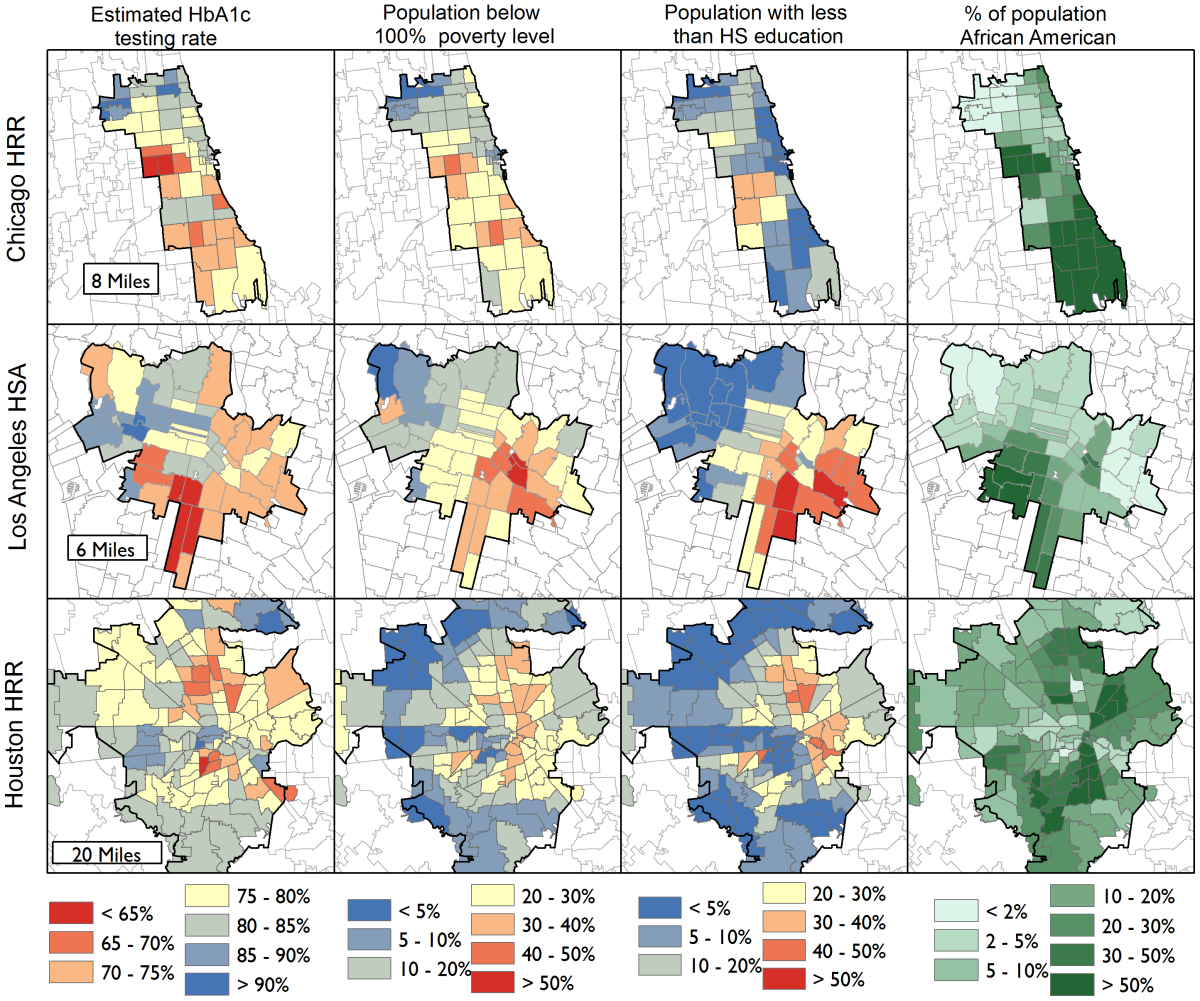
Regional Maps of ZIP Code-level Estimated Hemoglobin A1c Testing Rates Among Diabetic Medicare Beneficiaries, and General Population Demographic and Economic Characteristics.

We created several maps to visually explore national and regional testing rate variations and local population characteristics. Figure 2 presents the national map of ZIP code-level HbA1c testing rate estimates from 2009. For presentation purposes, the data are stylized as an elevation map; similar values are blended together, and transitions between “valleys” and “peaks” demarcated by gradations in color from red (lowest rates) to blue (highest rates). In Figure 3, we explore local variations within three major metropolitan regions: Chicago, Los Angeles, and Houston. In these maps, we have highlighted the ZIP codes that are located within regions commonly used for healthcare research: either HRRs (Chicago and Houston) or the smaller HSA (Los Angeles). Within each region, there are ZIP codes with very low and very high estimated HbA1c testing rates, as well as a wide range of population characteristics. The poorest ZIP codes, or those with the highest proportion of African-Americans, are not necessarily the ZIP codes with the lowest rates of HbA1c testing.

**Figure 4:**
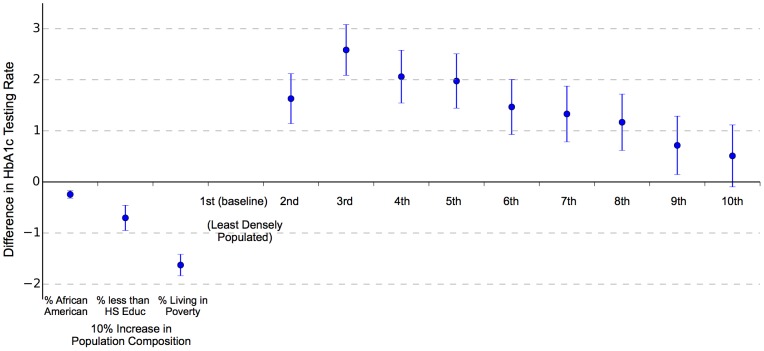
Multiple Regression Results: Hemglobin A1c Testing Rate Differences Associated With Population Characteristics. ZIP code-level HbA1c testing rates regressed on local population characteristics. Bars represent the 95% CI around the estimated association between each covariate and local HbA1c testing rates.

To make this comparison of sociodemographic factors and quality measures more formally, we performed a multiple linear regression at the ZIP code-level. Figure 4 presents the results. We regressed the raw rate from each ZIP code on the percent of the local population that was African American, the percent with less than a high school education, and the percent living below 100% FPL, as well as indicators for the decile of population density (with the least populated decile as reference). Each 10% increase in the percent of a ZIP's population that was African American was associated with a 0.24% decrease (95% CI −0.32–−0.17) in HbA1c testing rates. The corresponding values for 10% increases in the percent of the population that had less than a high school education or were living below 100% FPL were a 0.7% (95% CI −0.95–−0.46) decrease and a 1.6% decrease (95% CI −1.84–−1.42), respectively. The least densely populated ZIP codes had the lowest testing rates. Rates increased noticeably in the 2^nd^ and 3^rd^ most populated deciles, by 1.63% (95% CI 1.14–2.12) and 2.58% (95% CI 2.09–3.08), each relative to the first decile, and then decreased again with increasing population density. Testing rates in the 10^th^ (most densely populated) decile of ZIP codes were not significantly different from those in the 1^st^. The total r-squared from the regression was 0.048. In sensitivity analyses, our main findings were consistent when we used spatially smoothed HbA1c rates rather than raw rates, or when we restricted the sample to ZIP codes whose raw rates were based on larger populations (the r-squared did increase with these alternative approaches, particularly using the smoothed outcome variable, but was never higher than 0.09).

## Discussion

We document extensive fine-grained variations in HbA1c testing rates among Medicare beneficiaries. Local sociodemographic characteristics are related to testing rates, as the populations of ZIP codes with lower testing rates tend to be comprised of a greater proportion of residents living in poverty, with less than a high school education, or who are African American. Patients from extremely rural or urban ZIP codes may face additional barriers to care, perhaps due to the difficulty of accessing an adequate provider. Yet, all of these population characteristics explain less than 5% of the variation in testing rates, which can vary quite widely within relatively small geographical areas.

Our results have several implications for providers, and emerging ACOs in particular. First, the fact that demographic differences explain so little of the variation across ZIP codes is somewhat promising for the potential of ACOs – or any health system-based initiative – to improve healthcare quality, as it suggests that some providers are able to provide high quality care to vulnerable populations. This finding is also consistent with previous work showing relatively moderate disparities in healthcare quality among Medicare beneficiaries across ethnic and socioeconomic lines [25]. It should be difficult for providers to claim that they were unable to deliver high quality care due solely to the composition of their patient population.

Yet, place of residence does matter; in each of the regions we examined closely, there was at least one ZIP code whose testing rate was substantially lower than its neighbors'. Such hotspots cannot be explained entirely by identifiable population characteristics; they may also reflect idiosyncratic characteristics of place that affect residents' experience and result in barriers to care. Previous studies of healthcare quality and disparities have focused mainly on providers, and have demonstrated that minority patients are often concentrated among lower quality providers [8], [26]. To the extent that residents of a ZIP code are served by the same provider, our findings could reflect similar patient sorting. Yet it is likely that ZIP code-level measures reflect local social, cultural, or economic effects as well as the influence of the dominant healthcare system.

These ZIP code-based measures can be useful for rewarding high quality care. Penalizing providers for seemingly poor quality care that is beyond their control is unfair, and may exacerbate disparities if these providers are subsequently unable to expend resources on improving care [2], [5]. At the same time, excessively generous risk adjustment may only maintain disparities by giving providers a free pass to continue providing inadequate care simply because their patients have historically received lower quality care. Rather, incorporating some measure of improvement – that is, changes over time in quality measures for an assigned patient population – may help reconcile this dilemma [27]. An alternative option would be to offer extra incentives for providers to take on patients from hotspot ZIP codes and ensure that they receive adequate care.

Awareness of local fine-grained variations may also help serve as a surveillance system, helping providers track an important risk factor for barriers to care that may be overlooked in the clinical setting. Rather than retrospectively reviewing the care processes for patients who didn't receive HbA1c tests, providers could reach out to patients from regions with historically low testing rates and inquire about local barriers to care. For example, inadequate public transportation from specific areas could impede patients' access to testing sites during operating hours, but physicians are unlikely to discuss such barriers in a routine clinical exam. Another possible result of these inquiries is that providers may consider reaching out to local community groups in specific areas to develop innovative ways to improve care.

The spatial analysis approach we pursued in this paper allowed us to explore fine-grained variation within the regions (whether states, HRRs or HSAs) that are typically used for measuring healthcare services; we created a separate estimate for each ZIP code. We validated these estimates by comparing them to the raw rates observed in the 2010 data, and found very clear associations between the first year's spatially smoothed rates and the following year's raw data. If we had reported only the raw rates for the ZIP codes with at least 25 observations – a minimum often used for public reporting of quality data [24] – we would have been limited to reporting statistics for less than 20% of the ZIP codes in our original sample. Alternatively, aggregating data to larger areas, such as those highlighted in Figure 3, would have obscured substantial local variations.

Limitations to our findings include our reliance on Medicare claims data, which are a byproduct of the billing process. In some cases, payment mechanisms may mask evidence of clinical services. For example, beneficiaries who receive services through Federally Qualified Health Centers or Rural Health Centers (clinics intended to serve rural and/or indigent populations [28]) could have had their HbA1c tests ordered in these clinics. Yet, these clinics use a bundled payment mechanism; specific tests are not billed. For this reason, we excluded any beneficiary with a visit to one of these clinics, which may have biased our cohorts towards more wealthy, non-rural beneficiaries. If anything, this would likely bias our estimates upwards, especially in areas whose populations tend to rely on care from such providers.

A separate concern arises from the spatial analysis methods we used to create our ZIP code-level estimates. We chose a relatively straightforward approach, yet the literature suggests that our approach is well suited for geographic data representing populations of varying density [20]. Additionally, sensitivity analyses in which we performed regressions under a range of different assumptions confirmed our main findings. Finally, we used the ZIP code as our spatial unit because it is the smallest available area to which Medicare beneficiaries can readily be assigned. Providers can not be held responsible for a ZIP code, but this approach allowed us to gain insight into local factors that may affect patients' care.

## Conclusions

Local population characteristics are associated with HbA1c testing rates among resident Medicare diabetics, but demographic differences explain very little of the variation in testing rates. This result suggests that some providers are able to deliver high quality care to vulnerable populations. At the same time, residents from some ZIP codes appear to face greater access challenges than their neighbors, independent of the demographics of the local populations. Payers may want to consider rewarding providers for improving the care of patients from such areas, while ACOs should consider incorporating fine-grained geographic measures into their quality monitoring and improvement efforts.

## Supporting Information

Data S1 Comma-separated data file.
(TXT)

Codebook S1 Descriptions of each variable in the Data S1 file.
(TXT)
